# The Mechanisms Underlying the Hypolipidaemic Effects of *Grifola frondosa* in the Liver of Rats

**DOI:** 10.3389/fmicb.2016.01186

**Published:** 2016-08-03

**Authors:** Yinrun Ding, Chun Xiao, Qingping Wu, Yizhen Xie, Xiangmin Li, Huiping Hu, Liangqiu Li

**Affiliations:** ^1^School of Bioscience and Bioengineering, South China University of TechnologyGuangzhou, China; ^2^State Key Laboratory of Applied Microbiology Southern China, Guangdong Provincial Key Laboratory of Microbial Culture Collection and Application, Guangdong Open Laboratory of Applied Microbiology, Guangdong Institute of MicrobiologyGuangzhou, China; ^3^Department of Biology, Basic Medical College, Guangdong Medical UniversityZhangjiang, China

**Keywords:** *Grifola frondosa*, hyperlipidaemia, lipid metabolism, gene expression, two-dimensional electrophoresis, proteomics

## Abstract

The present study investigated the hypolipidaemic effects of *Grifola frondosa* and its regulation mechanism involved in lipid metabolism in liver of rats fed a high-cholesterol diet. The body weights and serum lipid levels of control rats, of hyperlipidaemic rats, and of hyperlipidaemic rats treated with oral *G. frondosa* were determined. mRNA expression and concentration of key lipid metabolism enzymes were investigated. Serum cholesterol, triacylglycerol, and low-density lipoprotein cholesterol levels were markedly decreased in hyperlipidaemic rats treated with *G. frondosa* compared with untreated hyperlipidaemic rats. mRNA expression of 3-hydroxy-3-methylglutaryl-CoA reductase (HMGCR), acyl-coenzyme A: cholesterol acyltransferase (ACAT2), apolipoprotein B (ApoB), fatty acid synthase (FAS), and acetyl-CoA carboxylase (ACC1) were significantly down-regulated, while expression of cholesterol 7-alpha-hydroxylase (CYP7A1) was significantly up-regulated in the livers of treated rats compared with untreated hyperlipidaemic rats. The concentrations of these enzymes also paralleled the observed changes in mRNA expression. Two-dimensional polyacrylamide gel electrophoresis (2-DE) and Matrix-Assisted Laser Desorption/Ionization Time of Flight Mass Spectrometry (MALDI-TOF-MS) were used to identify 20 proteins differentially expressed in livers of rats treated with *G. frondosa* compared with untreated hyperlipidemic rats. Of these 20 proteins, seven proteins were down-regulated, and 13 proteins were up-regulated. These findings indicate that the hypolipidaemic effects of *G. frondosa* reflected its modulation of key enzymes involved in cholesterol and triacylglycerol biosynthesis, absorption, and catabolic pathways. *G. frondosa* may exert anti-atherosclerotic effects by inhibiting LDL oxidation through down-regulation and up-regulating proteins expression in the liver of rats. Therefore, *G. frondosa* may produce both hypolipidaemic and anti-atherosclerotic effects, and potentially be of use as a functional food for the treatment or prevention of hyperlipidaemia and atherosclerosis.

## Introduction

Hyperlipidaemia is a metabolic disorder with a high global prevalence characterized by elevated circulating levels of total cholesterol (TC), low-density lipoprotein cholesterol (LDL-C), and triacylglycerols (TG), with a reduced concentration of high-density lipoprotein cholesterol (HDL-C) in the blood (Hongli et al., 2013). Hyperlipidaemia is a primary risk factor for the development of atherosclerosis, coronary heart disease, peripheral artery diseases, and other major metabolic diseases (Watts and Fredrik, 2011). It has been reported that even 1% reduce in the serum TC levels was estimated to result in 2–3% decrease in the risk of cardiovascular disease (Catapano et al., 2000). Therefore, the reduction of circulating TC and LDL-C levels are important strategy that can be employed to prevent or slow the progression of atherosclerosis (Hansson, 2005). Normal physiological functions can be maintained by decreasing elevated serum TC to an appropriate level. Research into functional foods has identified plants and fungi with beneficial effects in certain diseases (Mélanie and Jones, 2004); for example, a novel functional diet formulation of *Auricularia auricula* and Chinese hawthorn (*Crataegus pinnatifida)* showed effective hypolipidaemic properties (Luo et al., 2009). A large number of bioactive components of foods have been reported to potentially produce hypolipidaemic effects. These findings have intensified the search for foods that lower serum lipid levels effectively with minimum side effects (Liu et al., 2014).

*Grifola frondosa* is a promising candidate with lipids-lowering effects that may prevent atherosclerotic disease through its high fiber, microelement, protein, and polysaccharide content (Gu et al., 2007). *G. frondosa*, also known as Maitake, is a basidiomycete polypore fungus belonging to the Meripilaceae family (Shin and Lee, 2014). *G. frondosa* is a medicinal mushroom in traditional Chinese and Japanese herbology, as well as being widely used is an edible mushroom for cooking, and dietary supplementation in Asia. This fungus has shown various biological activities, including antitumor (Kodama et al., 2003), immuno-modulatory (Gary et al., 2009), hypotensive (Talpur et al., 2002), and antioxidant effects. Recently, an antioxidant polysaccharide was purified from *G. frondosa* (Chen et al., 2012), and our laboratory has previously demonstrated that *G. frondosa* polysaccharides produced hypoglycaemic effects in a mouse model of type 2 diabetes (Xiao et al., 2011). Therefore, *G. frondosa* can be considered to have potential as a functional food. The hypolipidaemic effects of *G. frondosa* were previously reported that it reduced serum lipid levels by promoting the conversion of cholesterol to bile acid (Kubo and Nanba, 1997). Subsequent investigations (Fukushima et al., 2001) showed that *G. frondosa* fiber significantly reduced serum lipid levels by enhancing fecal cholesterol excretion. Another study (Mayumi et al., 2013) also revealed that *G. frondosa* reduced serum lipid levels. However, few studies have investigated whether *G. frondosa* can regulate the expression of key genes involved in lipid metabolism.

Hepatic cholesterol metabolism is an important physiological process involved in lipid metabolic disorder (Li et al., 2014). Cholesterol homeostasis is regulated by coordinated changes in cholesterol biosynthesis, absorption, catabolism, and transport of lipoprotein particles. HMGCR is a key rate-limiting enzyme for *de novo* cholesterol synthesis. ACAT2 is a key tissue cholesterol-esterifying enzyme that plays an key role in cholesterol absorption by catalyzing the formation of cholesterol esters from cholesterol and long-chain fatty acids *in vivo* in response to excess intracellular cholesterol (Lee et al., 2005). CYP7A1 is a key rate-limiting enzyme for bile acid biosynthesis from cholesterol (Hubacek and Bobkova, 2006). Approximately 95% of LDL is ApoB, which is constitutively expressed in the liver. ACC1 is a key enzymes in the synthesis of fatty acids and triacylglycerol in the liver. And few research has been carried out concerning whether the lipid-lowering effects of *G. frondosa* are regulated by these key genes. Therefore, this study was undertaken to assess the lipid-lowering effects of *G. frondosa* and its regulation mechanism on key genes involved in lipid metabolism in liver of rat, i.e., HMGCR, ACAT2, ApoB, CYP7A1, FAS, and ACC1.

## Materials and methods

### Sample preparation

Dried *G. frondosa* fruiting bodies were obtained from Guangdong Yuewei Edible Fungi Technology Co. Ltd (Guangzhou, China). After drying with hot air at 40°C for 3 h, the dehydrated fruiting bodies were milled to a powder with a particle size of ~0.3 mm using a grinding and mixing machine (BFM-6A; Ji'nan Supertime Technology Co. Ltd, Jinan, China). The dried *G. frondosa* fruiting body powder was reconstituted in purified water at a ratio of 1:13 (w/v). A dose of 760 mg/kg body weight administered to rats based on our laboratory health care product studies of the hypolipidaemic effect of *A. auricular*.

### Animals, diets, and hyperlipidaemia

Thirty male 8-week-old specific pathogen-free Sprague-Dawley rats (weighing 200 ± 20 g) were purchased from Guangdong Medical Laboratory Animal Center, Guangzhou, China (production certificate no. scxk [Yue] 2011-0015; quality certificate no. 44002100002251; Experimental Animals License no. syxk [Yue] 2013-0011). The rats were housed individually in stainless steel cages in a specific pathogen-free room at a controlled temperature (22 ± 2°C) with relative humidity of 55 ± 5% and a 12 h cycle of light and dark. All animal procedures complied with the Guide for the Care and Use of Laboratory Animals and were approved by the Animal Care Committee of the Center for Disease Control and Prevention (Guangdong Province, China).

After acclimatization for 7 days, the rats were randomly divided into two groups: a control group (*n* = 10) and a hyperlipidaemic group (*n* = 20). Control rats were provided with a basal diet (quality certificate no. 4420030005388) and the hyperlipidaemic group was provided with a hypercholesterolaemic diet (quality certificate no. 4420030000055). The basal diet consisted of 33.64% corn, 22.5% bran, 17% flour, 17.5% soybean meal, 1.33% soybean oil, 4% fish meal, 1% powder, 2% dicalcium phosphate, 1% additive, and 0.03% choline. The hypercholesterolaemic diet consisted of 52.2% basal diet, 1.2% cholesterol, 20% sucrose, 15% lard oil, 10% casein, 0.6% calcium bicarbonate, 0.2% sodium cholate, 0.4% mountain flour, and 0.4% premix.

After 2 weeks, a blood sample was collected from the tail vein of each rat and assayed for serum TC levels using a Hitachi 7060 automatic biochemical analyzer (Hitachi Limited, Tokyo, Japan). Serum TC levels were markedly elevated (*P* < 0.05) in the hyperlipidaemic group compared with the control group. All 20 rats receiving the hypercholesterolaemic diet for 2 weeks exhibited hyperlipidaemia. The hyperlipidaemic rats were then randomly divided into two groups: a hyperlipidaemic group and a *G. frondosa* group.

The control rats continued to receive basal diet and were orally administered with distilled water at the same time every morning for 5 weeks. The hyperlipidaemic group received the hypercholesterolaemic diet and were orally administered with distilled water at the same time every morning for 5 weeks. The *G. frondosa* group received the hypercholesterolaemic diet and were orally administered with 760 mg/kg body weight *G. frondosa* at the same time every morning for 5 weeks.

### Sampling

At the end of the experimental period, all rats were sacrificed (without fasting). Blood samples were collected into heparinized tubes using a disposable plastic syringe and left at room temperature to coagulate for 20 min. Serum was obtained by centrifugation at 3000 g for 10 min at 4°C and stored at −70°C until use. Livers were removed, washed with normal saline, and blotted dry on filter paper before snap-freezing in liquid nitrogen and storage at −70°C until further analysis.

### Serum biochemical analysis

Serum levels of TC, TG, LDL-C, and HDL-C were determined using the Hitachi 7060 automatic biochemical analyser and commercial assay kits (Bio Sino Bio-technology and Science Inc), according to the manufacturer's instructions.

### Enzymatic concentration assay

The enzymatic concentrations of HMGCR, ACAT2, CYP7A1, ApoB, FAS, and ACC1 in rat liver samples were determined using appropriate rat enzyme-linked immunosorbent assay (ELISA) kits (Westan Biotech Co), according to the manufacturer's instructions, and using a Thermo Scientific microplate reader (Multiskan Go).

### RNA preparation, cDNA synthesis, and quantitative real-time PCR (RT-PCR)

Total RNA was prepared using RNAiso Plus (Takara, code no. 9108) according to the manufacturer's protocol. Total RNA was transcribed into cDNA using a Prime Script TM RT Master Mix (Takara, code no. RR036A) according to the manufacturer's instructions. RT-PCR was carried out on a Master cycle Rep Realplex RT-PCR system (Eppendorf, Germany) using SYBR Premix Ex Taq (Tli RNase H Plus; Takara, Code No. RR420A) to determine the expression of HMGCR, ACAT2, ApoB, CYP7A1, FAS, and ACC in rat liver tissue. Glyceraldehyde-3-phosphate dehydrogenase (GAPDH) was used as an internal control. The primers for RT-PCR are presented in Table 1. PCR was carried out in duplicate by incubating the reaction mix at 95°C for 2 min followed by 40 cycles of 95°C for 15 s, 60°C for 20 s, and 72°C for 30 s. The relative levels of target mRNAs were determined using the 2^−ΔΔCt^ method and were normalized to the GAPDH mRNA signals in each sample.

**Table 1:** **SYBR green primer sequences used for RT-PCR**.

**Gene**	**Accession No**.	**Forward primers (5′ to 3′)**	**Reverse primers (5′ to 3′)**	**Size(bp)**
GAPDH	NM017008.4	CCGCATCTTCTTGTGCAGTG	TCCCGTTGATGACCAGCTTC	250
HMGCR	XM006231826.1	GAGCGTTCGTGGGTCCAG	GGCACAACCTGCCGTATCTA	242
ACAT2	NM_153728.2	GAACGTGGTGGTCCATGACT	TTCAGCAGACCTCCAACCAC	201
CYP7A1	NM_012942.2	TGGAGAACGGGTTGATTCCG	CTGTGTCCAAATGCCTTCGC	235
ApoB	NM019287.2	AGCTGATCGAAGTGTCCAGC	TGTTAACCGCATGGCTCAGT	246
FAS	NM017332.1	CCACAGGACAAGCCCATCTT	TCGGAGACAGTTCACCAAGC	159
ACC1	NM022193.1	ACAACGCAGGCATCAGAAGA	GCTGTGCTGCAGGAAGATTG	245

### Protein preparation, 2-DE, and protein identification

Rat livers were individually pulverized in liquid nitrogen and dissolved in 1 ml alklysis buffer containing 40 mM Tris base, 7 M urea, 2 M thiourea, 4% CHAPs, 65 mM dithiothreitol, and 2% IPG buffer. The solution was centrifuged at 12,000 rpm for 10 min at 4°C and supernatant was purified using a Ready Prep 2-D Clean-up Kit (catalog no. 163-2130, BioRad) according to the manufacturer's protocol. A non-interference protein assay kit (Sang on Biotech Co. Ltd, SK3071) was used to determine the protein concentrations.

Protein samples (800 μg) were mixed with rehydration buffer (containing IPG buffer, pH 4–7) and passively rehydrated overnight (12–16 h) at ambient temperature. In the first-dimension isoelectric focusing phase, proteins were focused sequentially at 100 V for 30 min, 250 V for 1 h, 1000 V for 2 h, 4000 V for 2 h, 10,000 V for 3 h, 60,000 V. h and finally kept at 500 V. The strips were equilibrated twice for 15 min in the first and second equilibration buffers and transferred onto a 12% polyacrylamide gel for second-dimension electrophoresis, which was performed at 100 V, 30 A for 30 min followed by 200 V, 150 A for 8 h. All gels were then silver-stained.

The stained 2-DE gels were scanned using the Image Master 2D platinum 5.0 software (GE Healthcare China, Guangzhou), which was also used to match and analyze the images. Spots with a ≥1.5-fold change in intensity were analyzed to determine the statistical significance of any differences in protein expression between the study groups. The protein spots of interest were excised from the gels and washed twice with MilliQ water. The gel slices were digested at 37°C overnight (16 h) with sequencing grade modified trypsin. The digested samples were identified using MALDI-TOF-MS. Protein identification was conducted using Mascot software and the Uniprot rat data base. The differential proteins were then loaded to the Kyoto Encyclopedia of Genes and Genomes (KEGG) Mapper (www.genome.jp/kegg/), and an in-house Perl script was used to identify pathway over-representation.

### Statistical analysis

All results are expressed as the mean ± standard deviation (SD). Differences between groups were determined by one-way ANOVA. Statistical differences were considered significant at *P* < 0.05. SPSS software version 21.0 was used for all analyses.

## Results

### Effect of *Grifola frondosa* on body weight

**Figure 1:**
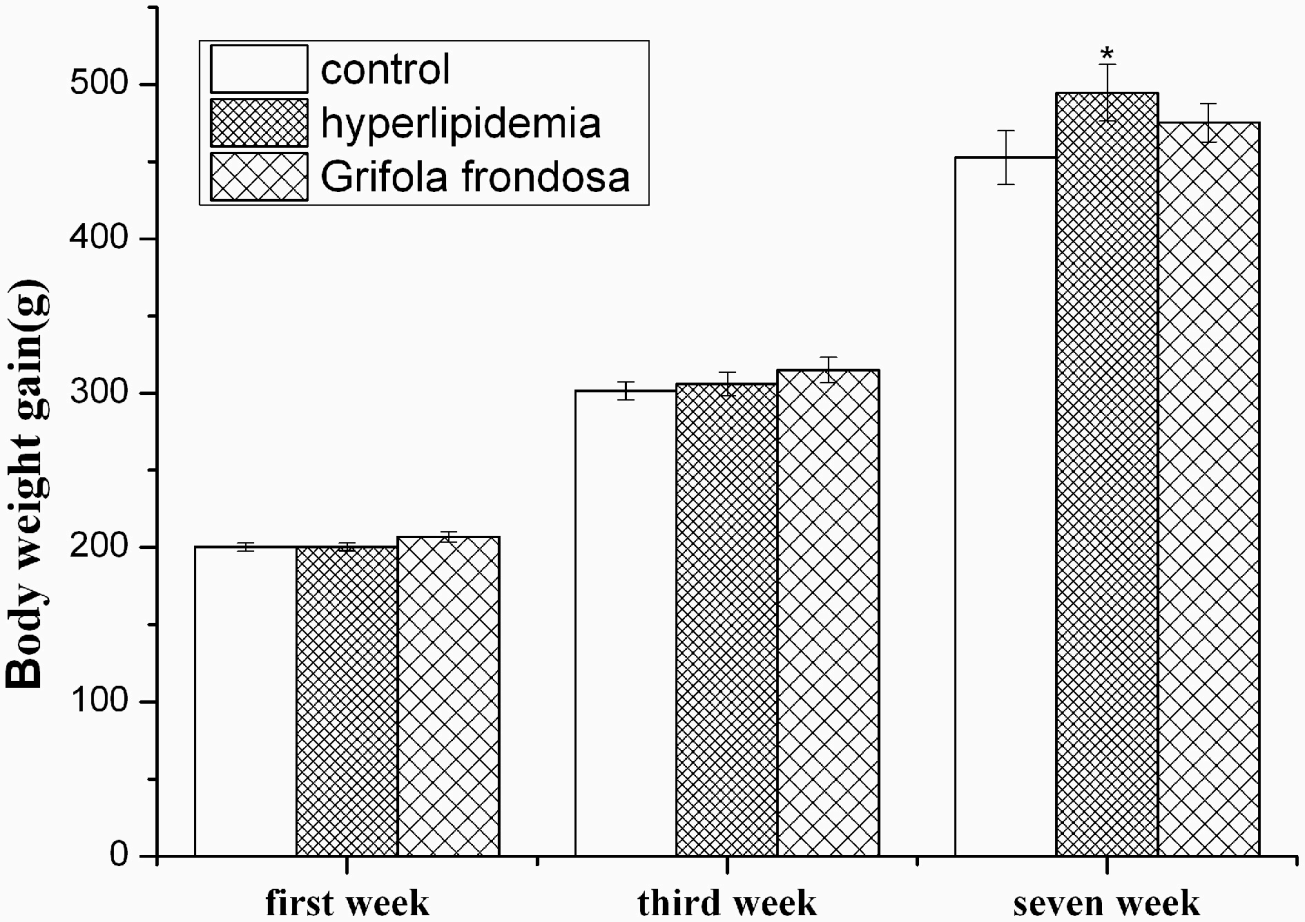
Effect of *Grifola frondosa* on body weight (^*^P < 0.05, control group vs. hyperlipidemic group).

Rat body weight data are shown in Figure 1. After acclimatization for 1 week and the establishment of hyperlipidemic for another 2 weeks, there were no significant differences in body weight observed between the control, hyperlipidemic, and *G. frondosa* groups. At the end of the experiment (7 weeks), the results indicated that hyperlipidemic rats significantly increased in body weight compared with the control group (^*^P < 0.05), but no significant differences in body weight gain were observed between the hyperlipidemic and *G. frondosa* groups.

### Effects of *Grifola frondosa* on rat serum lipid levels

**Figure 2:**
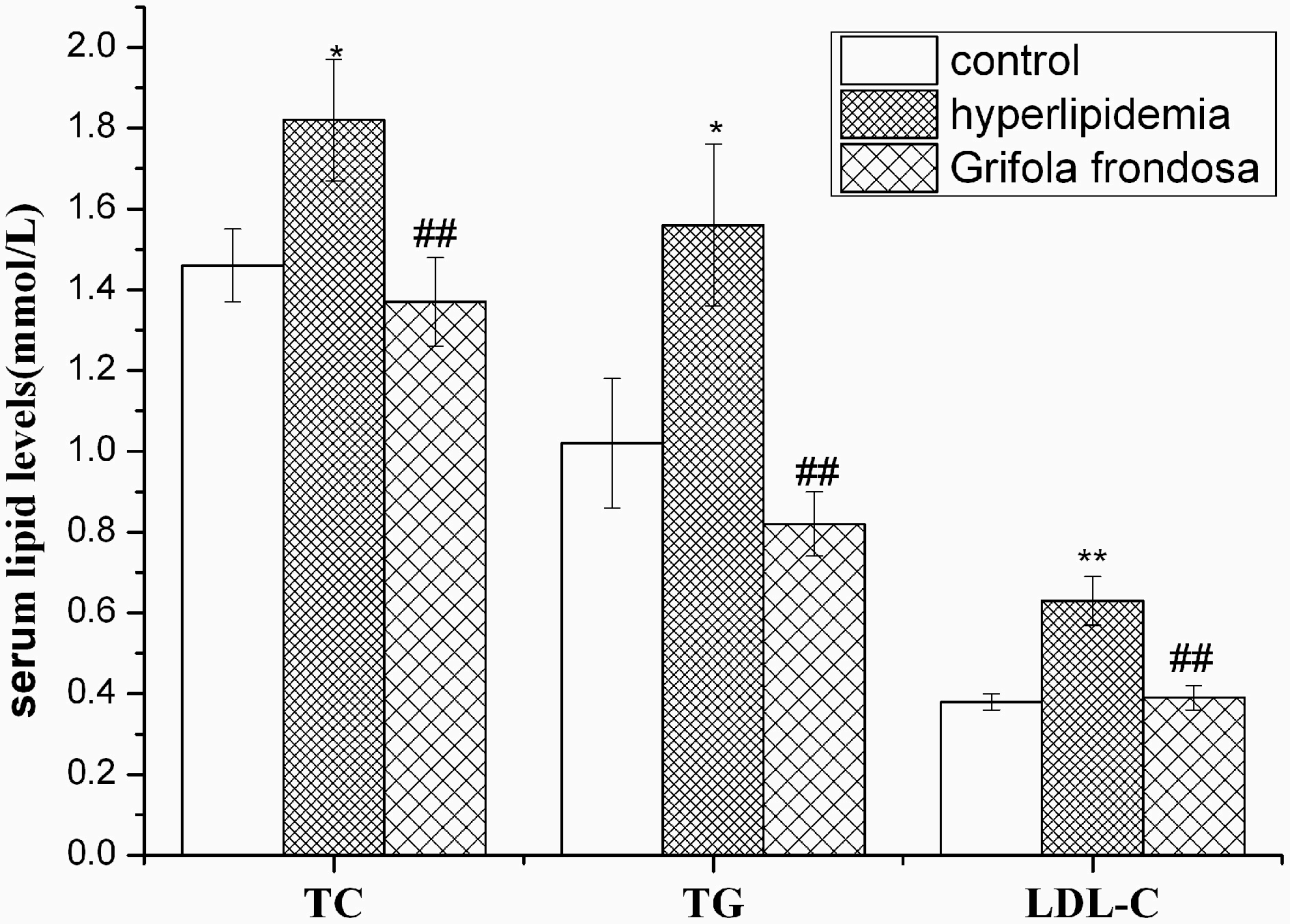
Effects of *Grifola frondosa* on serum lipid levels of rats (^*^P < 0.05, ^**^P < 0.01, control group vs. hyperlipidemic group; ^#^*P* < 0.05, ^#^^#^*P* < 0.01, *G. frondosa* group vs. hyperlipidemic group).

As summarized in Figure 2, serum TG, TC, and LDL-C levels were markedly elevated in hyperlipidaemic rats compared with the control group. Administration of *G. frondosa* led to significant reductions in serum TG, TC, and LDL-C levels compared with those of the hyperlipidaemic group, indicating the beneficial effects of *G. frondosa* treatment on serum lipid profiles in hyperlipidemic rats.

### Effects of *Grifola frondosa* on rat liver expression of key genes involved in lipid metabolism

**Figure 3:**
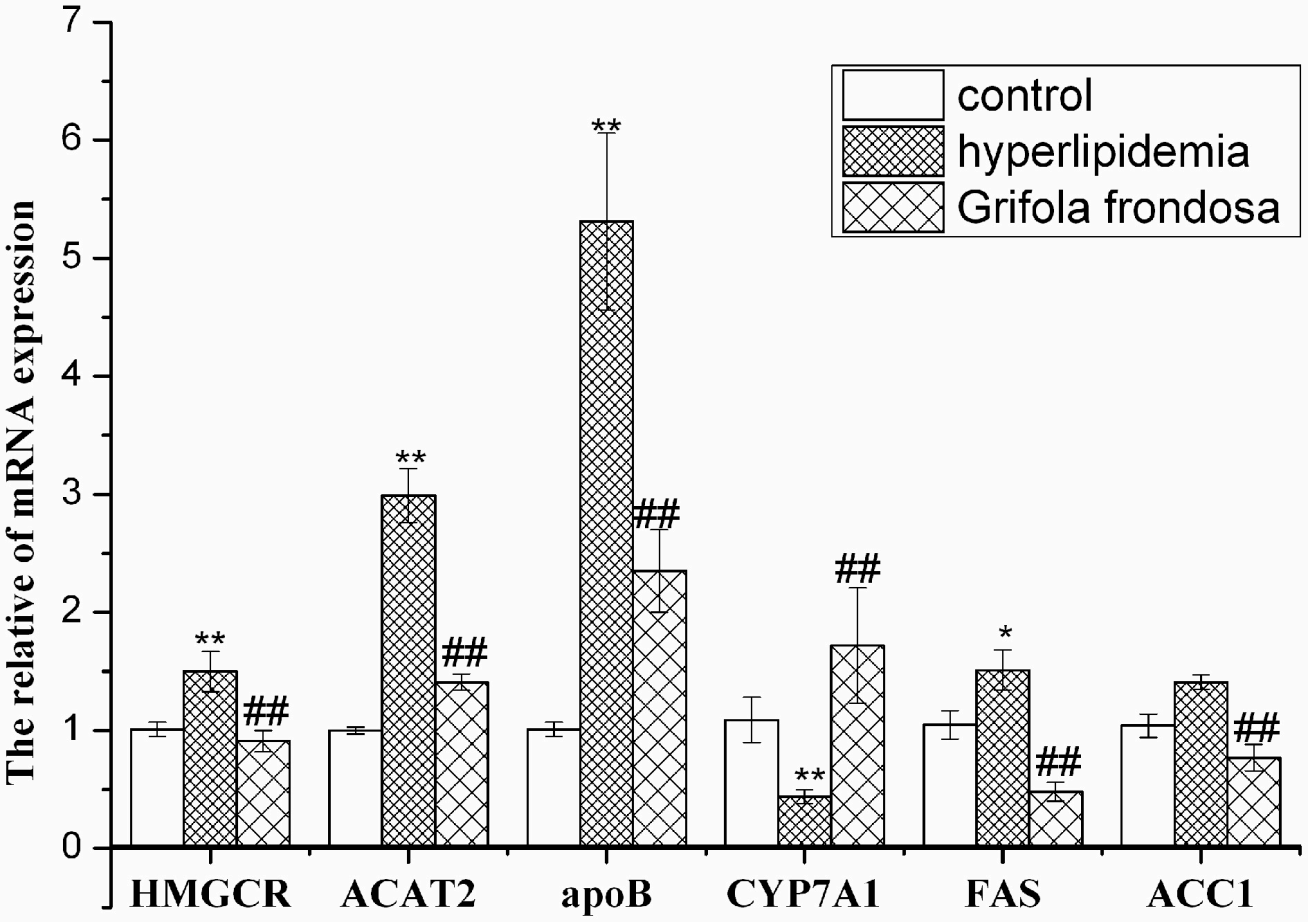
Effects of *Grifola frondosa* on mRNA expression of key genes in rat liver (^*^P < 0.05, ^**^P < 0.01, control group vs. hyperlipidemic group; ^#^*P* < 0.05, ^#^^#^*P* < 0.01, *G. frondosa* group vs. hyperlipidemic group).

RT-PCR data (Figure 3) showed that liver HMGCR, ACAT2, ApoB, and FAS mRNA expression levels were elevated in hyperlipidaemic rats compared with the control group, but were markedly decreased in hyperlipidaemic rats treated with *G. frondosa*. Although ACC1 gene expression was not up-regulated in hyperlipidaemic rat liver compared with the control group, it was significantly reduced in hyperlipidaemic rats treated with *G. frondosa*. In contrast, liver CYP7A1 gene expression was down-regulated in hyperlipidaemic rats compared with the control group, but significantly increased in hyperlipidaemic rats following *G. frondosa* administration.

### Effects of *Grifola frondosa* on the concentration of key enzymes involved in lipid metabolism in rat liver

The ELISA data (Table 2) showed that same trends in liver concentrations of HMGCR, ACAT2, ApoB, CYP7A1, and FAS enzymes reflected the expression of their respective mRNAs. Notably, liver ACC1 enzyme concentration was significantly increased in hyperlipidaemic rats compared with the control group, but significantly reduced in rats treated with *G. frondosa*.

**Table 2:** **Effects of *Grifola frondosa* on enzymatic concentration of rat liver**.

**Enzyme**	**Control**	**Hyperlipidemia**	***Grifola frondosa***
HMGCR(U/L)	4.73 ± 1.55	14.56 ± 0.23^**^	10.68 ± 0.26^##^
ACAT2(U/L)	56.81 ± 10.84	155.41 ± 9.90^**^	89.16 ± 12.39^##^
ApoB(μg/ml)	551.22 ± 85.24	1007.95 ± 73.21^**^	732.89 ± 85.00^##^
CYP7A1(U/L)	47.32 ± 1.58	20.13 ± 3.85^**^	35.97 ± 1.26^#^
FAS (nmol/L)	7.06 ± 0.69	16.28 ± 0.88^**^	13.15 ± 0.51^##^
ACC1 (pmol/L)	393.29 ± 23.58	774.48 ± 29.36^**^	575.09 ± 18.52^##^

** P < 0.01, control group vs. hyperlipidemic group;

# P < 0.05,

## *P < 0.01, Grifola frondosa group vs. hyperlipidemic group*.

### 2-DE image and protein identification by MALDI-TOF-MS

**Figure 4:**
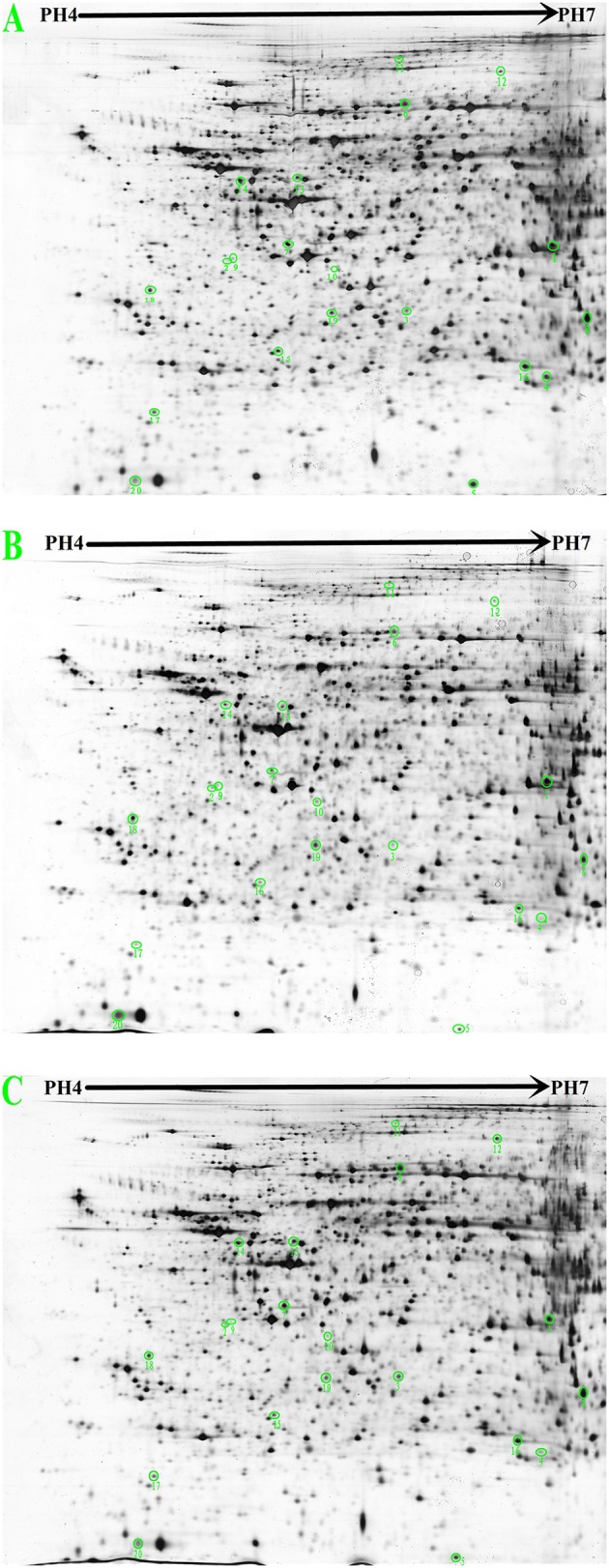
**2-DE images from rat liver protein extracts (proteins were separated in the first dimension at pH 4.0–7.0 and on a 12% SDS-PAGE gel in the second dimension. (A)** Control group. **(B)** Hyperlipidemic group. **(C)** *Grifola frondosa* group).

Figure 4 shows the 2-DE images obtained from rat liver protein extracts and illustrates the locations of differentially expressed spots. Approximately 1400 protein spots were identified on the 4–7 NL range gels by silver staining. Spots with a ≥1.5-fold change in intensity were analyzed. Differential analysis showed that 98 spots were up-regulated in the control group compared with the hyperlipidaemic group and 80 spots were up-regulated in the *G. frondosa* group compared with the hyperlipidaemic group. Twenty-three repeat spots were present in both of these differentially regulated groups. Furthermore, 59 spots were down-regulated in the control group compared with the hyperlipidaemic group and 74 spots were down-regulated in the *G. frondosa* group compared with the hyperlipidaemic group. 17 repeat spots were present in both of these differentially regulated groups. Therefore, these 40 (23 + 17) repeat protein spots were chosen for analysis by MALDI-TOF-MS and 20 of these were identified (Figure 4 and Table 3).

**Table 3:** **Identified rat liver proteins**.

**No**.	**Accession No**.	**Gene name**	**Protein name**	**Mascot score**	**Sequence coverage (%)**	**MW (Da)**	**PI**	**Biological function**	**Protein expression(*fold change*)**
1	HMCS2_RAT	Hmgcs2	Hydroxy methylglutaryl-CoA synthase, mitochondrial	96	19	57,332	8.86	This enzyme condenses acetyl-CoA with acetoacetyl-CoA to form HMG-CoA, which is the substrate for HMG-CoA reductase	↓
2	ACSL1_RAT	Acsl1	Long-chain-fatty-acid–CoA ligase 1	253	16	79,155	6.60	Activation of long-chain fatty acids for synthesis of cellular lipids	↓
3	AMACR_RAT	Amacr	Alpha-methylacyl-CoA racemase	126	21	42,201	6.38	This enzyme is required for the beta-oxidation of branched-chain fatty acids	↑
4	PRDX1_RAT	Prdx1	Peroxiredoxin-1,Prdx-1	239	51	22,323	8.27	Eliminates peroxides generated during metabolism; elevation of Prdx-1 levels reduces atherosclerotic lesion progression	↑
5	SODC_RAT	Sod1	Superoxide dismutase [Cu-Zn]	229	42	16,073	5.88	Destroys radicals normally produced within the cells which are toxic to biological systems	↑
6	HEMO_RAT	Hpx	Hemopexin	215	25	52,060	7.58	Haem binding protein significantly decreased the activity of lipid peroxidation	↑
7	METK1_RAT	Mat1a	S-adenosylmethionine synthase isoform type-1	201	26	44,240	5.61	Catalyzes the formation of S-adenosylmethionine from methionine and ATP	↑
8	BHMT1_RAT	Bhmt	Betaine–homocysteine S-methyltransferase 1	265	32	45,404	8.02	Converts betaine and homocysteine to dimethylglycine and methionine	↑
9	CH60_RAT	Hspd1	60 kDa heat shock protein, mitochondrial	209	15	61,088	5.91	Expression of HSP60 was positively correlated to the degree of atherosclerosis, HSP60 may be the initiating factor for atherosclerosis	↓
10	CPPED_RAT	Cpped1	Serine/threonine-protein phosphatase CPPED1	120	23	35,581	5.40	Inhibitors of serine/threonine protein phosphatase for treatment or prevention of arteriosclerotic diseases	↓
11	CPSM_RAT	Cps1	Carbamoyl-phosphatesynthase, mitochondrial	150	10	1,65,673	6.33	Catalyzes synthesis of carbamoyl phosphate from ammonia and bicarbonate	↑
12	SARDH_RAT	Sardh	Sarcosine dehydrogenase, mitochondrial	333	23	10,2573	6.15	Sarcosine degradation; synthesizes formaldehyde and glycine from sarcosine	↑
13	ACTG_RAT	Actg1	Actin, cytoplasmic 2	350	45	42,108	5.31	Actins are highly conserved proteins, ubiquitously expressed in all eukaryotic cells	↑
14	K1C18_RAT	Krt18	Keratin, type I cytoskeletal 18	355	32	47,732	5.17	When phosphorylated, plays a role in filament reorganization	↑
15	CATB_RAT	Ctsb	Cathepsin B	229	23	38,358	5.36	Participates in intracellular degradation and turnover of proteins	↑
16	ATPA_RAT	Atp5a1	ATP synthase subunit alpha, mitochondrial	356	27	59,831	9.22	Mitochondrial membrane ATP synthase (F1F0 ATP synthase or complex V) produces ATP from ADP	↑
17	PSB9_RAT	Psmb9	Proteasome subunit beta type-9	100	24	23,424	4.88	Cleavage of peptide bonds with very broad specificity	↑
18	TPM3_RAT	Tpm3	Tropomyosin alpha-3 chain	210	39	29,217	4.75	Binds to actin filaments in cells; in non-muscle cells, is implicated in stabilizing cytoskeleton actin filaments	↓
19	GSH0_RAT	Gclm	Glutamate–cysteine ligase regulatory subunit	127	24	30,871	5.36	Involved in the sub-pathway that synthesizes glutathione from L-cysteine and L-glutamate	↓
20	CYB5_RAT	Cyb5a	Cytochrome b5	248	34	15,346	4.90	Electron carrier for several membrane-bound oxygenases	↓

*↑: Protein expression up-regulated in control and G. frondosa groups compared with the hyperlipidemic group*.

*↓: Protein expression down-regulated in control and G. frondosa groups compared with the hyperlipidemic group*.

The 20 differentially expressed proteins were identified by searching the Mascot Uniprot rat database for the peptide mass fingerprint. Eight of which were relevant to lipid metabolism. 3-hydroxyl-3-methylglutaryl-CoA synthase 2 (HMGCS2) is an important enzyme involved in cholesterol synthesis. Long-chain-fatty-acid-CoA ligase 1 (ACSL1) is an important enzyme in synthesis of fatty acids (Yen et al., 2008). alpha-methylacyl-CoA racemase (AMACR) is required for the beta-oxidation of branched-chain fatty acids (Mobley et al., 2003). Five of eight proteins are Peroxiredoxin-1 (Prdx-1), superoxide dismutase (SOD1), haemopexin (HPX), 60 kDa heat shock protein (HSP60), and serine/threonine-protein phosphatase (CPPED1), all of which are related to lipid peroxidation and atherosclerosis. The 12 of 20 proteins are implicated in other metabolic pathways, 5 of 12 proteins are relevant to amino acid metabolism, S-adenosylmethionine synthase isoform type-1 (MAT1A) catalyzes the formation of S-adenosylmethionine from methionine. glutamate-cysteine ligase regulatory subunit (GCLM) is involved in the sub pathway that synthesizes glutathione from L-cysteine and L-glutamate. betaine-homocysteine S-methyltransferase 1 (BHMT) converts betaine and homocysteine to dimethylglycine and methionine. carbamoyl-phosphatesynthase (CPS1) catalyzes the synthesis of carbamoyl phosphate from ammonia and bicarbonate. sarcosine dehydrogenase (SARDH) catalyzes the synthesis of formaldehyde and glycine from sarcosine. Three of twelve proteins are actin (ACTG1), keratin, type I cytoskeletal 18 (KRT18) and tropomyosin alpha-3 chain (TPM3), all of which are related to cytoskeleton stability. cathepsin B (CTSB) and proteasome subunit beta type-9 (PSMB9) are related to protein degradation. ATP synthase subunit alpha (ATP5a1) and proteasome subunit beta type-9 (PSMB9) are relevant to energy metabolism. Details of the 20 proteins that showed a ≥1.5-fold change in density ratio are provided in Figure 4 and Table 3.

## Discussion

Hypercholesterolemia and hypertriglyceridemia are important risk factors that act, either alone, or in combination, to accelerate the development of cardiovascular disease and the progression of atherosclerotic lesions (Hun et al., 2007). Thus, reducing serum TC and/or TG levels are important for atherosclerosis and cardiovascular disease prevention. High levels of LDL accumulate in the extracellular subendothelial space of arteries to form oxidized LDL, which is highly atherogenic and toxic to vascular cells. It is generally accepted that lowering high serum LDL levels plays an important role in the prevention and reversal of atherosclerosis (Haiping et al., 2010). Diet plays a key role in the control of cholesterol balance, and the consumption of a high-cholesterol diet is regarded as a crucial risk factor for the development of hyperlipidaemia. In our study, the rats ingesting a high-cholesterol diet developed elevated serum levels of TG, TC, and LDL-C, which were significantly reduced by the administration of *G. frondosa*. The results also agreed with those of previous studies that *G. frondosa* significantly reduced serum TG, TC, and LDL-C levels (Kubo and Nanba, 1997; Mayumi et al., 2013). These findings suggest that *G. frondosa* is a potentially effective lipid-lowering food. Individuals with hyperlipidemia could consider using *G. frondosa* as a dietary supplement for preventing atherosclerosis and cardiovascular diseases. However, these studies didn't elaborate whether the lipid-lowering effects of *G. frondosa* are mediated by key genes and proteins involved in lipid metabolism. Therefore, we investigated its mechanisms on the hepatic metabolic pathway of lipid by evaluating key enzyme expression. Furthermore, we elucidated changes in liver proteomic profiles using 2DE combined with MALDI-TOF-MS.

The inhibition of HMGCR expression leads to suppression of cholesterol synthesis in liver and further efficiently reduce serum cholesterol level (Lee et al., 2003). Therefore, HMGCR is a key target of several widely available cholesterol-lowering drugs. Statins, currently the most widely used hypolipidaemic drugs, reduce serum TC levels by inhibiting hepatic HMGCR expression (Lammi et al., 2015). A quantity of edible mushroom such as *Pleurotus ostreatus, Cratharellus cornucopiodes, Amanita ponderosa*, and particularly *Lentinus edodes* showed HMGCR inhibitory capacities ranging from 52 up to 76% *in vitro* (Gil-Ramírez et al., 2013). *A. auricular* significantly decreased HMGCR activity in mice liver (Chen et al., 2011). Our study show that *G. frondosa* potentially capable of down-regulating the mRNA expression of HMGCR and markedly reduced serum TC levels in rats. These results showed the inhibitors of HMGCR represents the most efficient way to reduce serum TC levels. Accumulation of hepatic cholesteryl esters promote potentially hyperlipidemia. Tissue-specific knockouts of ACAT2 prevent diet induced hepatic cholesteryl esters and TC accumulation in the liver and blood of mice (Zhang et al., 2012). Inhibition of ACAT2 expression may reduce the levels of plasma TC and accumulation of cholesterol ester on the arterial wall (Bei et al., 2012). In current study, *G. frondosa* reduce the accumulation of serum TC of rats by down-regulating the mRNA expression of ACAT2. Therefore, the ACAT2-specific inhibitors are effective in treating or preventing hyperlipidemia. ApoB is an essential component of LDL and inhibition of ApoB synthesis in the liver may efficiently reduce the serum LDL. Mipomersen decreases ApoB synthesis by inhibiting translation of ApoB mRNA (Thomas et al., 2013). *G. frondosa* decreased ApoB synthesis by reducing content of ApoB mRNA and further reduce serum LDL level. The increase of CYP7A1 expression may enhance catabolic of cholesterol and thus reduce the serum TC level. *L. edodes* may contribute to reducing TC, LDL, TG, hepatic fat accumulation and aortic atherosclerotic plaque formation by increasing mRNA expression of CYP7A1 in liver tissues (Yang et al., 2013). In our study, administration of *G. frondosa* enhanced CYP7A1 mRNA expression, resulting in increase of cholesterol conversion into bile acids.

Cholesterol homeostasis is regulated by well-balanced mechanisms controlling mRNA encoding multiple enzymes (Kenichiro et al., 2005). The concentrations of these enzymes in the present study also paralleled the observed changes in mRNA levels. Down-regulation of HMGCR, ACAT2, and ApoB mRNA expression by administering *G. frondosa* reduced the concentration of these enzymes and may have resulted in the decreased serum TC and LDL levels. Conversely, up-regulation of CYP7A1 mRNA expression by administering *G. frondosa* increased concentration of the enzyme and may promote the conversion of cholesterol to bile acid, thus reducing serum TC levels. Therefore, cholesterol-lowering effects of *G. frondosa* are regulated by down-regulation of HMGCR, ACAT2, and ApoB and up-regulation of CYP7A1 levels, which inhibited the synthesis, absorption and transport of cholesterol, and enhanced its catabolism.

Accumulation of TG in hepatocytes is associated with metabolic disorder (Fatiha et al., 2013). ACC1 is a key rate-limiting enzyme in synthesis of fatty acids and TG in the liver. ACC1 catalyzes the carboxylation of acetyl-CoA to form malonyl-CoA, while FAS catalyzes palmitate formation (Menendez et al., 2009). The inhibition of ACC1 expression will limit TG *de novo* synthesis and further reduce serum TG levels. Gemfibrozil, currently widely used hypotriglyceridemic drugs, reduce serum TG levels by inhibiting hepatic ACC1 expression. We found significant reductions in the mRNA expression levels and concentration of FAS and ACC1 in rats treated with *G. frondosa*, as well as lower serum TG levels. Consequently, *G. frondosa* may inhibit FAS and ACC1 expression to produce beneficial effects in hypertriglyceridaemia, thus reducing serum TG and risk of cardiovascular disease.

To further investigate the influence of *G. frondosa* on liver lipid metabolism, we employed proteomic tools (2-DE and MALDI-TOF-MS) to globally identify proteins that were differentially expressed in the livers of rats administered with *G. frondosa* compared with untreated hyperlipidemic rats. Our proteomic data revealed that 20 proteins were differentially expressed between the two groups. Interestingly, HMGCS2 is relevant synthesis of cholesterol and condenses acetyl-CoA with acetoacetyl-CoA to form 3-hydroxy-3-methylglutaryl-CoA (HMGC), which is the substrate for HMGCR. HMGCR is a key rate-limiting enzyme in cholesterol synthesis and catalyzes the production of mevalonic acid from HMGC. Down-regulation of HMGCS2 protein expression (Table 3) may inhibit cholesterol biosynthesis and further decrease serum TC levels. The marked reduction in serum TC levels observed in rats administered *G. frondosa* could be explained by the down-regulation of HMGCS2 protein expression. Among the 20 identified proteins, ACSL1 and AMACR are of interest with respect to fatty acid metabolism. ACSL1 catalyzes the conversion of free fatty acids to acyl–CoA and the reaction of acyl–CoA with diacylglycerol to form TG. Similar investigations (Ying-Ling et al., 2014) showed that toll-like receptor agonists promote prolonged TG storage in murine and human macrophages by enhancing fatty acid uptake and increasing TG synthesis, with an associated persistent increases in ACSL1. Therefore, down-regulation of ACSL1 protein expression could inhibit fatty acid biosynthesis. The other protein, AMACR, is required for oxidation of fatty acids (Mobley et al., 2003), and it's up-regulation could enhance catabolism of fatty acids. The inhibition of ACSL1, required for fatty acid biosynthesis, and the increase in AMACR, involved in fatty acid catabolism, could therefore result in decreased fatty acid content and further decrease serum TG levels in rats treated with *G. frondosa*.

High levels of LDL are present in the vessel walls, and LDL undergoes oxidative attack by reactive oxygen species (ROS) to generate oxidized LDL (oxLDL). Endothelial cell dysfunction and the accumulation of oxLDL in the vessel wall are early events in atherogenesis, resulting in the recruitment of circulating monocytes, their adhesion to the endothelium, their subsequent differentiation into macrophages, and the accumulation of oxLDL to form foam cells. Therefore, oxLDL plays a pivotal role in the initiation and promotion of atherosclerosis (Augusti et al., 2012), and lowering oxLDL levels represents an important strategy for the treatment and prevention of atherosclerosis. SOD and Prdx-1 both are member of a ubiquitous family of antioxidant enzymes that reduces oxLDL levels and early atherosclerosis. SOD destroys radicals that are toxic to biological systems. Tea polyphenols may confer anti-atherosclerotic effects by inhibiting oxidation of LDL by SOD (Uto-Kondo et al., 2013). The elevated Prdx-1 level associated with T-cell death-associated gene 51 deficiency contributes to increased resistance to oxidative stress, leading to reduce atherosclerotic lesion growth in mice (Hossain et al., 2013). HPX has a strong binding capacity to haem and thus can transport free toxic haem. HPX also exhibits anti-oxidant, immune regulation, and organic protection effects. When bound to HPX, haem significantly reduced oxygen free radical levels and decreased the catalytic activity of lipid peroxidation (oxLDL) (Grinberg et al., 1999). Therefore, up-regulation of SOD1, Prdx-1, and HPX expression can increase resistance to oxLDL formation, thereby reducing atherosclerotic lesion progression. CPPED1 dephosphorylates AKT (protein kinase B) family kinases, specifically at Ser-473. Studies (Vaittinen et al., 2013) demonstrated that knockdown of CPPED1 expression enhances insulin-stimulated glucose uptake in mature adipocytes, leading to improved glucose metabolism via adiponectin-mediated mechanisms. The results (Walter, 2006) indicated that inhibitors of CPPED1 may be useful for the treatment or prevention of arteriosclerotic diseases. These results showed down-regulation of CPPED1 expression prevent arteriosclerotic disease. In response to free radicals and oxidative stress, cells produce high levels of HSP60. Studies (Yu et al., 2013) showed that *Porphyromonas gingivalis* challenge increased the expression of oxidative stress-related mediators, such as CCL2 and HSP60. In contrast, consumption of green tea epigallocatechin-3-gallate decreased atherosclerotic lesions and oxidative stress-related mediators in atherosclerotic mice induced by *P. gingivalis*. Overall, down-regulation of CPPED1, and HSP60 reduces atherosclerotic lesion progression. To summarize, we showed that *G. frondosa* produced anti-atherosclerotic effects by inhibiting LDLox formation via a reduction CPPED1 and HSP60 levels and increased levels of SOD1, Prdx-1, and HPX in rat liver.

Eight of the 20 proteins identified were relevant to lipid metabolism. The remaining 12 proteins are implicated in other metabolic pathways, MAT1A, GCLM, BHMT, CPS1, and SARDH are relevant to amino acid metabolism. MAT1A form S-adenosylmethionine (SAM), which is precursor of glutathione (GSH). GCLM is an important enzyme in synthesis of GSH, which has antioxidation effect. Therefore, MAT1A and GCLM indirectly involved in lipid peroxidation. ACTG1, KRT18, and TPM3 keep cytoskeleton stability to provide support frame for lipid metabolism. CTSB and PSMB9 degrade excess lipoprotein in cell. ATP5a1 and Cyb5a provide energy for lipid metabolism.

## Conclusions

**Figure 5:**
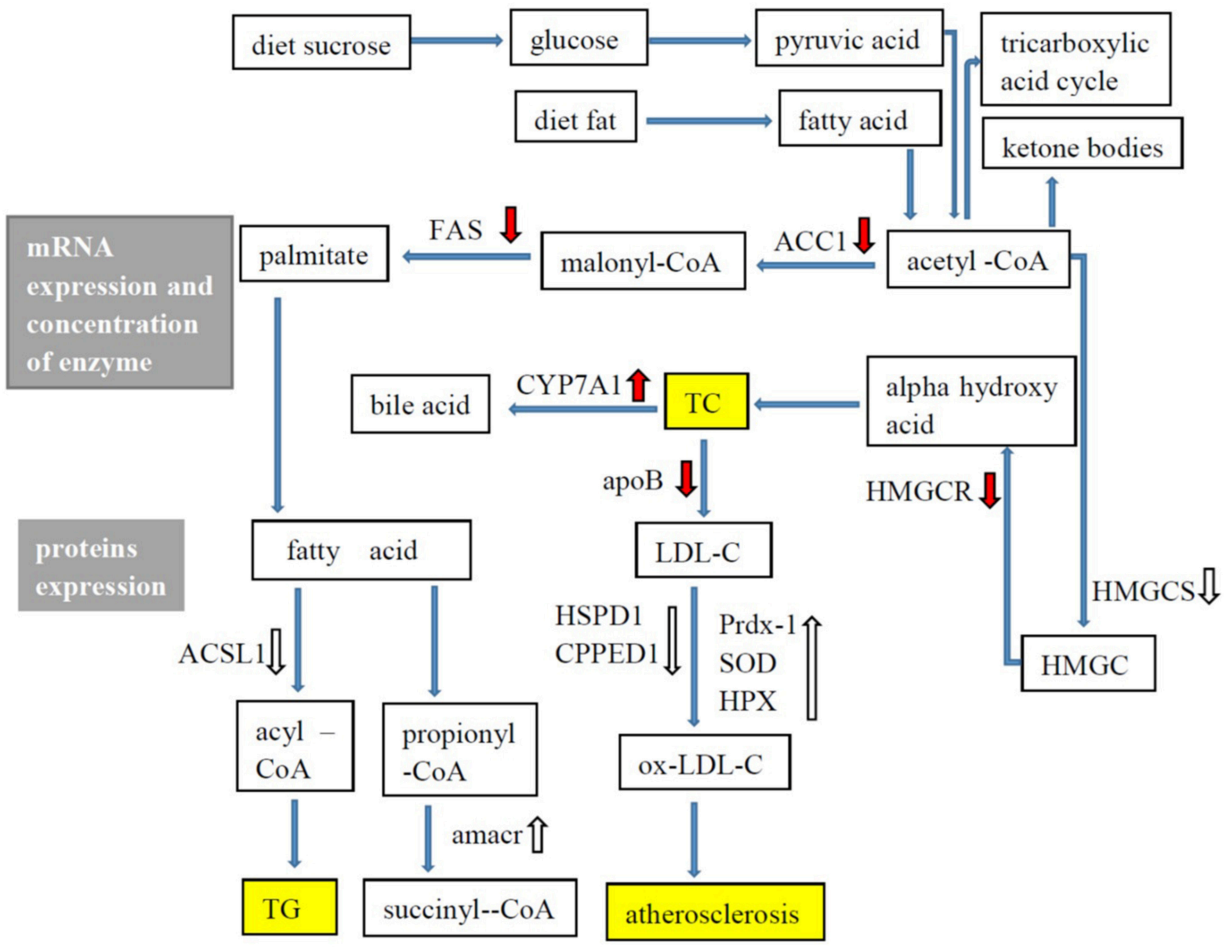
**Lipid metabolism in rat liver**. 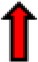: mRNA expression, enzymatic concentration up-regulated in *Grifola frondosa* group compared with the hyperlipidemic group. 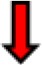: mRNA expression, enzymatic concentration down-regulated in *G. frondosa* group compared with the hyperlipidemic group. 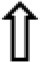: Protein expression up-regulated in *G. frondosa* group compared with the hyperlipidemic group; 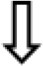: Protein expression down-regulated in *G. frondosa* group compared with the hyperlipidemic group.

*G. frondosa* exerted hypolipidaemic and anti-atherosclerotic effects in this study. Figure 5 shows the hypolipidaemic effects of *G. frondosa* through down-regulation of HMGCR, HMGCS2, ACAT2, and ApoB and up-regulation of CYP7A1 expression levels of mRNA or proteins involved in cholesterol biosynthesis, absorption, catabolism. Furthermore, *G. frondosa* down-regulated FAS, ACC1, and ACSL1 and up-regulated AMACR expression levels of mRNA or proteins involved in triacylglycerol biosynthesis and catabolism. The anti-atherosclerotic effects of *G. frondosa* can function through the inhibition of LDL oxidation by down-regulated HSP60 and CPPED1 and up-regulated Prdx-1, SOD1, and HPX expression levels in rat liver. These findings indicate that *G. frondosa* has significant potential as a functional food to treat or prevent hyperlipidaemia and atherosclerosis.

## Author contributions

YD and QW designed the experiments; YD, CX, XL, and HH performed the experiments; YD and YX performed data analysis; LL provided scientific expertise; YD wrote the manuscript; QW edited the manuscript.

### Conflict of interest statement

The authors declare that the research was conducted in the absence of any commercial or financial relationships that could be construed as a potential conflict of interest.

## Abbreviations

TC: total cholesterol
TG: triacylglycerol
LDL-C: low-density lipoprotein-cholesterol
HDL-C: high-density lipoprotein-cholesterol
HMGCR: 3-hydroxy-3-methylglutaryl-CoA reductase
ACAT2: acyl-Coenzyme A: cholesterol acyltransferase
CYP7A1: Cholesterol 7-alpha-hydroxylase, Cytochrome P450, family 7, subfamily a, polypeptide 1
ApoB: apolipoprotein B
FAS: fatty acid synthase
ACC1: Acetyl-CoA carboxylase
HMGCS2: 3-hydroxyl-3-methylglutaryl-CoA synthase 2
HMGC: 3-hydroxy-3-methylglutaryl-CoA
ACSL1: long-chain-fatty-acid–CoA ligase 1
HSPD1: 60 kDa heat shock protein
CPPED1: serine/threonine-protein phosphatase
AMACR: alpha-methylacyl-CoA racemase
Prdx-1: Peroxiredoxin-1
SOD: Superoxide dismutase
HPX: Haemopexin
RT-PCR: real-time PCR
GAPDH: Glyceraldehyde-3-phosphate dehydrogenase
2-DE: two-dimensional polyacrylamide gel electrophoresis
MALDI-TOF-MS: Matrix-Assisted Laser Desorption/ Ionization Time of Flight Mass Spectrometry.
